# Simultaneous Eye Tracking and Cerebral Hemodynamic Monitoring in Infants: A Guide for Pediatric Outpatient Follow-Up

**DOI:** 10.3390/brainsci15050469

**Published:** 2025-04-28

**Authors:** Valéria Azevedo de Almeida, Maria Clara Lima da Cruz, Nicole Rodrigues Morais, Italo Vinicius Tavares Rodrigues, Cintia Ricaele Ferreira da Silva, Edgard Morya, Silvana Alves Pereira

**Affiliations:** 1 Alberto Santos Dumont Institute of Education and Research (ISD), RN, Macaíba CEP 59288-899, Brazil; valeria@edu.isd.org.br (V.A.d.A.); claralimadc@gmail.com (M.C.L.d.C.); edgard.morya@isd.org.br (E.M.); 2 Physiotherapy Department, Federal University of Rio Grande do Norte, RN, Natal CEP 59078-970, Brazil; nicole.morais.700@ufrn.edu.br (N.R.M.); italo.rodrigues.123@ufrn.edu.br (I.V.T.R.); cintia.silva@edu.isd.org.br (C.R.F.d.S.)

**Keywords:** infants, newborn, model of visual recognition, eye-tracking technology, low- and middle-income countries, country, low income

## Abstract

Simultaneous eye tracking and cerebral hemodynamic monitoring contribute to the understanding of neural responses to stimuli in infants. However, exploring the impact of complex socioeconomic and environmental adversities on neurodevelopment requires transitioning this tool from research laboratories into clinical practice to evaluate its feasibility in outpatient contexts. Background/Objectives: This study aimed to present a protocol for simultaneously integrating functional near-infrared spectroscopy (fNIRS) with eye tracking (ET) in infants at risk for neurodevelopmental disorders in a clinical setting with limited resources, during a cognitive task. Methods:The protocol was applied to infants in their first 12 months of life. The infants were exposed to tasks involving the processing of social and non-social stimuli, while their brain signals were monitored using fNIRS and their eyes were tracked with ET. The protocol included three main stages: (1) pre-collection, involving the preparation and habituation of the infants and equipment setup (fNIRS and ET); (2) cognitive function monitoring, using social and non-social stimuli to assess preferential processing via fNIRS and ET; and (3) post-collection, with guidelines for data pre-processing and analysis. Results: The application of the protocol allowed for the identification of technical challenges and the adaptation of procedures for clinical use. The main methodological challenges were difficulty using the conventional cap, excessive movement, synchronization issues between fNIRS and ET, and difficulties calibrating both devices across different age groups. Conclusions: The standardization proposed in this protocol enables healthcare professionals to explore different neurocognitive aspects in pediatric clinical settings and expands the scope of neurodevelopmental assessments.

## 1. Introduction

Neurodevelopment begins in prenatal life and extends into the early years after birth, occurring most intensely until the age of two or three years [1]. During this period, the brain is particularly sensitive to environmental influences, whether positive or negative [1]. Interest in monitoring child development has grown, driven by technological advances that enable more objective and accurate assessments [2,3,4,5].

During the first year of life, infants undergo significant cognitive changes, marked by the maturation of visual attention, memory, perception, and problem-solving abilities [6]. This phase is characterized by intense neural reorganization, facilitated by brain plasticity and interaction with the environment [7]. From the earliest months, infants already demonstrate visual preferences, such as increased interest in human faces and social stimuli, as well as developing skills like object permanence and visual categorization [8,9,10].

To understand neurocognitive development in infants, it is necessary to use assessment techniques that are applicable in economic, cultural, and educational contexts, enabling the early identification of infants at risk for long-term cognitive deficits [11,12,13]. Traditionally, this process was mainly based on standardized instruments such as the Bayley Scales of Infant and Toddler Development, the Mullen Scales of Early Learning, or the Denver Developmental Screening Test, which provide a global estimate of the child’s motor, language, and cognitive abilities. Although widely used, these tools have limitations, such as reduced sensitivity to detect subtle changes in cognitive processing and a strong dependence on behavioral performance in structured settings. Additionally, these tools are limited by the requirement for manual test administration and the subjective judgment of child behavior. They also generally require prolonged testing times, which makes large-scale standardization difficult and increases the likelihood of errors when assessing specific neurocognitive processes [14,15].

In this context, functional near-infrared spectroscopy (fNIRS) combined with eye-tracking (ET) technology has been gaining attention in studies with infants [14,15], and is considered safe for infants and young children [7,9].

fNIRS is a non-invasive optical neuroimaging technique that allows the measurement of changes in the concentration of hemoglobin in its oxygenated (oxyhemoglobin, HbO2) and deoxygenated (deoxyhemoglobin, HbR) forms in the brain tissue following neuronal activation [16,17]. The regular activity of neurons is sustained by energy produced from glucose and oxygen. An increase or decrease in neuronal activity alters the metabolic demand, resulting in a variation in oxygen consumption in the brain regions recruited during a task [18,19].

Eye tracking, on the other hand, allows for the extraction of data related to visual attention, providing information about how children interact with various visual stimuli [20,21,22]. The combination of these two techniques aims to understand the relationship between visual attention and brain function. It allows for the correlation of gaze direction patterns with changes in cerebral oxygenation in response to various stimuli [23].

The isolated use of either fNIRS or eye tracking presents limitations. While fNIRS provides information about neural correlates, it does not ensure that the infant is paying attention to the stimulus. Eye tracking, on the other hand, offers detailed data on attention but does not reveal how the brain processes that information.

From this perspective, this study aimed to integrate fNIRS and eye tracking simultaneously to overcome the limitations of using each method in isolation. By combining behavioral and neurophysiological measures in real time, it becomes possible to validate the neural response based on the infant’s actual visual attention to the stimulus. This represents a significant advancement over traditional approaches, which often assume that the child is engaged with the stimulus without an objective means of confirmation.

However, both ET and fNIRS present challenges when applied to infants and nonverbal children [9,24]. Eye tracking studies in pediatric populations often feature small sample sizes and limited metrics, making translation to clinical outpatient practice difficult [24]. Additionally, appropriate fNIRS data processing protocols are needed, particularly for correcting motion artifacts [9,25]. These challenges highlight the importance of addressing methodological issues to ensure data quality in pediatric studies [25,26].

Eye tracking (ET) has been widely used to assess aspects of infant cognition, such as visual preferences, fixation time on social and non-social stimuli, anticipation of events, and pattern-based learning [27,28]. Protocols such as Visual Paired Comparison and Preferential Looking Paradigms are used to measure recognition memory and stimulus discrimination, while gaze tracking is used to assess attentional control and cognitive flexibility [29,30,31]. Integrating these data with fNIRS enables the correlation of gaze patterns with changes in brain oxygenation, offering deeper insight into infant cognition [8,32].

However, although fNIRS and ET have significantly expanded in developmental research, few studies provide detailed protocols for applying these technologies to infants. Most research focuses on adults or older children due to the complexity and technical challenges of using these tools in infants. These challenges include frequent infant movement, difficulty calibrating fNIRS and ET devices for this age group, and concerns about the infant’s comfort and sensitivity during data collection [9,15,16,17,18,19,20,21,22,23].

A study presented a protocol developed to identify and optimize factors affecting fNIRS data quality collected from 6-to-9-month-old infants during a visual stimulation paradigm. The study reported hemodynamic data from 83% of the infants, highlighting the importance of ensuring optical contact and refining data rejection criteria [7].

Eye tracking can also be applied in cognitive load studies, where gaze direction and fixation duration are analyzed alongside fNIRS data to understand mental workload during tasks [7]. Additionally, the combined use of these tools has been explored to investigate the relationship between environmental factors and neurodevelopment, such as the impact of socioeconomic adversity and early experiences on infants’ cognitive development [9,10,11,33]. These technologies were implemented in a longitudinal protocol to assess neurocognitive development in infants in rural Gambia, alongside a parallel study in the UK. The use of a single protocol in a low-resource setting is promising and highlights the potential of these methods for similar contexts worldwide [5]. Such approaches have the potential to detect early developmental changes, including delays in visual attention or difficulties in stimulus discrimination, allowing for timely interventions during periods of heightened brain plasticity and responsiveness [24]. Translating these tools from research laboratories to clinical practice and evaluating their feasibility in infants at high risk for neurodevelopmental delays—particularly in low-resource settings—is a necessary step before examining associations between neurodevelopment and external factors.

In response to this need, the present study provides guidelines for the simultaneous integration of fNIRS and ET in infants in low-resource clinical settings. The protocol aims to investigate cognitive aspects from the first month of life, contributing to neurocognitive care across various healthcare professionals. Methodologies and guidelines are outlined for the following stages: pre-collection (infant preparation and desensitization, selection of ET and fNIRS equipment); cognitive function monitoring (preferential processing of social and non-social stimuli) using fNIRS and ET; and post-collection (pre-processing strategies and data analysis). Furthermore, perspectives, challenges, and limitations in collecting fNIRS and ET data from high-risk infants in low-resource clinical settings are discussed.

## 2. Materials and Methods

The protocol consists of a clinical assessment of overall development and the evaluation of cognitive function, specifically the preference for social and non-social stimuli in infants during the first year of life. The protocol was tested on infants of both sexes, of appropriate gestational age, in pediatric clinical care. These infants experienced early life adversities, including extreme poverty, malnutrition, recurrent infections, low maternal education, and maternal comorbidities.

Participants were recruited through direct invitation to parents or legal guardians during outpatient consultations. The inclusion criteria were infants aged up to 12 months, with no history of neurological or psychiatric disorders, and exhibiting expected cognitive and motor development for their age. Participants needed to tolerate being seated for a reasonable period and maintain adequate attention during experiments. Additionally, parents or guardians were required to read and sign the free informed consent form to authorize the infant participation. The exclusion criteria were infants with a clinical history of neurological disorders, uncorrected vision impairments, or significant difficulties in mobility or social interaction. Infants who did not tolerate the placement of the fNIRS cap or presented excessive discomfort during eye tracking calibration were also excluded from the study.

In addition to the exclusion criteria, infants with loss of fNIRS signals during data collection—caused by excessive movement or sensor displacement—were excluded from analysis. fNIRS signal loss occurs when optodes lose adequate scalp contact, compromising the quality of changes measured in the concentration of oxy- and deoxyhemoglobin. Similarly, loss of ET signals during calibration or data collection, due to excessive eye or head movement or technical tracker failure, also resulted in data exclusion.

The protocol included three main stages: (1) pre-collection, involving the preparation and habituation of the infants, as well as the setup of the equipment (fNIRS and ET); (2) cognitive function monitoring via fNIRS and ET using social and non-social stimuli to assess preferential processing; and (3) post-collection with guidelines for data pre-processing and analysis.

### 2.1. Pre-Collection: Preparation and Habituation of the Infants and Equipment Setup (fNIRS and ET)

#### 2.1.1. Pre-Collection

fNIRS is a non-invasive method to measure brain activity by acquiring changes in the concentration of oxy- and deoxyhemoglobin. The equipment used was the NIRSport 1 (NIRx Medical Technologies, Berlin, Germany) with 8 infrared emitters and 8 light sensors. These optodes were positioned approximately 2 cm apart and carefully coupled to the right and left frontotemporal, right and left parietal, and occipital regions (according to 10–20 system), covering the cortical areas relevant for analyzing brain activity related to the task. The montage of the optodes was adjusted according to the size of each infant’s head, to ensure the quality and accuracy of data collection. The signal intensity and the range of the emitters and sensors were adjusted for each region of interest, allowing the measurement of variations in brain oxygenation with accuracy.

**Figure 1:**
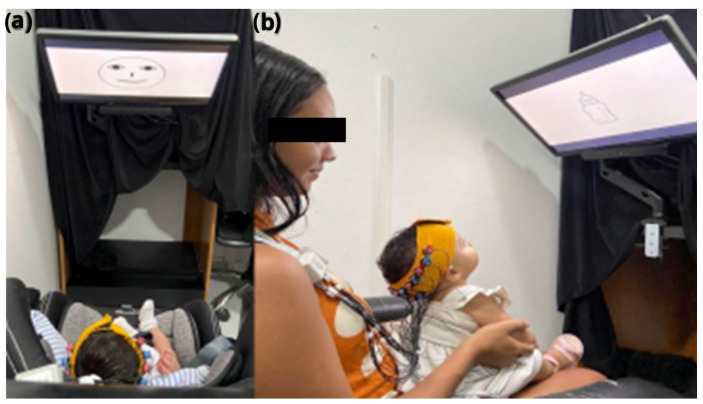
(**a**) Infant on the infant car seat in front of the screen showing a social stimulus, and (**b**) an infant held by the caregiver. In both cases, the screen was adjusted to a comfortable posture for the infant, allowing them to stare at it.

The Eye-tracker VT3 (Eye Tech Digital, Berlin, Germany) was coupled underneath a 22-inch screen, held by an articulated support, and positioned about 60 cm away from the infant’s eyes (on the seat or mother’s lap—Figure 1). The ET has a sampling rate of 200 Hz and an accuracy of 0.5°, allowing for detailed and highly accurate measurement of eye movements. The equipment’s accuracy is also guaranteed by its ability to detect up to 10 points of instability in eye tracking. To maintain the infants’ interest during the procedure, the points of instability were replaced by sequential visual animations displayed on the screen. The Mangold Vision 2021 software (Mangold International GmbH, Arnstorf, Germany) was used to present the visual stimuli on the screen, and to acquire and analyze eye tracking data. This setup allowed for accurate correlation of eye movement with oxyhemoglobin data acquired by fNIRS, enabling an integrated study of visual behavior and cognitive function in infants.

#### 2.1.2. Preparation for the Experiment

For data collection, the researcher comfortably positioned the infant in front of the screen on the infant seat or on the mother’s lap to minimize excessive head movement and avoid possible errors in the analysis, which could affect the calibration process and the dataset (Figure 1). The optodes and optical fibers have to be without obstacles and free to avoid low coupling. Caregivers were instructed to avoid soothing or rhythmic movements of the infant.

#### 2.1.3. Infant Habituation

Before the start of the experiment, infants underwent gradual preparation and habituation procedures to facilitate the use of the fNIRS and ET devices (including habituation to wearing the cap) in the outpatient clinic. This habituation phase involved gently placing the fNIRS cap on the infant, allowing them to become accustomed to the sensation of the device and minimizing discomfort during the experiment. The habituation phase was performed in a relaxed manner, ensuring that the infants felt comfortable before the start of the experimental trials.

**Figure 2:**
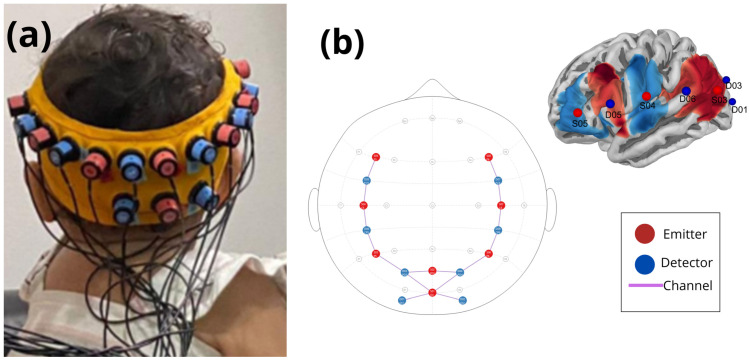
(**a**) Location of the eight emitters (red) and eight detectors (blue) over the occipital and parietal cortex. The optical fibers are free to allow head movement without dislocating the optodes or annoying the infant; (**b**) optode array projected on 3D brain surface showing changes in oxyhemoglobin, and the 2D optode montage according to the standard 10–20 system. Emitters are marked as red circles, detectors as blue circles, and lines indicate channel locations.

Proper positioning of the cap was essential, as incorrect positioning could affect signal quality and data reliability. We observed that infants younger than 4 months of age did not tolerate the conventional fNIRS cap, often crying when the cap was set with straps under their jaw. To address this issue, and following standard recommendations [12,13,21,34], we replaced the cap with a custom-made Velcro strap. The Velcro strap was made of a silicone band to keep the optode montage in position for three sizes of infant head circumferences: size S for head circumferences between 12.59 and 14.17 in, size M for 14.56 to 16.53 in, and size L for 16.92 to 18.7 in. Channel mapping adhered to NIRS registration aligned with magnetic resonance imaging (MRI), following the standard 10–20 system [21,34,35] (Figure 2).

The Velcro strap was placed on the infant after the optodes were mounted to optimize fit and reduce head manipulation during the assessment. In addition, it was placed before the appointment to help the infant become familiar with the strap pressure and touch. Spring-loaded grommets were avoided due to concerns about excessive pressure of the optode on the infant’s scalp, and infant toys were used to prevent an aversive sensation during the procedure.

#### 2.1.4. Eye Tracking Calibration

**Figure 3:**
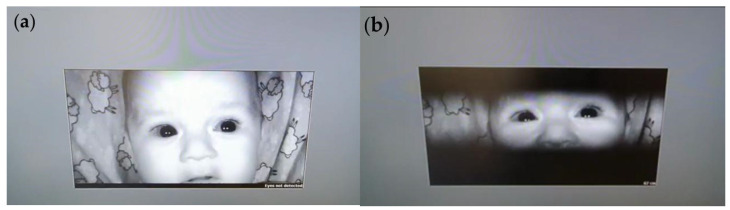
Illustrative screenshots during head positioning in front of the eye-tracker. The eye tracker software shows if the infant’s eyes (**a**) are not detected, allowing infant head repositioning to detect (**b**) the eyes for tracking automatically.

The ET calibration process started with the display of fixed calibration dots on the screen, followed by sequential visual animations designed to maintain the infant’s attention. This strategy allowed the system to continuously collect accurate data even during brief periods when fixation could not be precisely tracked. Calibration dots appeared at various positions on the screen to help the ET device calibrate the infant’s eye movements relative to visual stimuli. The ET device detected the position of the infant’s pupil and line of sight, enabling the software to adjust tracking accuracy automatically (Figure 3).

During calibration, the sensitivity of the ET device was also adjusted, enabling detection of eye movements under various lighting conditions and accommodating both small, rapid movements and larger, slower movements. The software automatically adapted sensitivity parameters to capture even subtle movements accurately.

### 2.2. Monitoring Cognitive Function Using Social and Non-Social Stimuli to Assess Preferential Processing via fNIRS and ET

**Figure 4:**
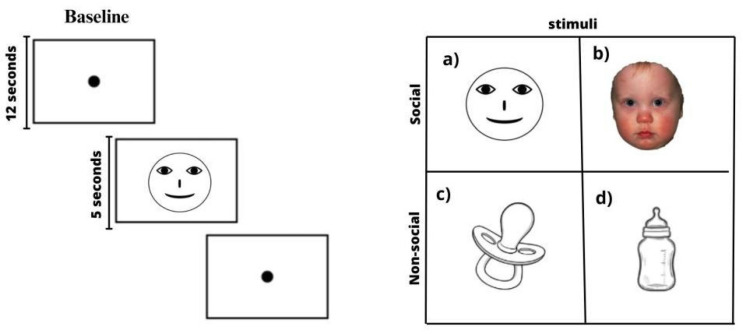
(**a**,**b**) Social and (**c**,**d**) non-social stimuli were displayed sequentially on the monitor for five seconds each. A 12 s interstimulus interval using a neutral background image with a black circle in the center was shown as a baseline.

Stimuli were presented sequentially, without randomization, in a predetermined order, on a high-definition monitor for five seconds each. The stimuli were organized into two main categories: social and non-social. Social stimuli consisted of two images: a graphic representation of a human face (eyes aligned above the nose and mouth) and a color photograph of an infant’s face. Non-social stimuli consisted of two neutral images of objects—an infant bottle and a pacifier—presented without emotional or social context. Social and non-social stimuli were shown in a fixed sequence, separated by 12 s intervals. During these intervals, a neutral background image with a central black circle was displayed to regain the child’s attention and allow the hemodynamic response to return to baseline, as illustrated in Figure 4.

The total duration of each task cycle was calculated based on four stimuli presentations (5 s each) and three intervals (12 s each), resulting in a total of (4 stimuli × 5 s) + (3 intervals × 12 s) = 20 s + 36 s = 56 s per complete cycle. Including calibration of both devices, the entire experimental session lasted approximately 10 to 15 min.

Considering that our experiment analyzed hemodynamic activity related to social and non-social stimuli, we used ET information to assess the behavior related to fNIRS recordings synchronized with the figures displayed on the screen. For this purpose, an fNIRS synchronization device was used, with triggers that marked the beginning and end of each stimulus. The eye tracking data were aligned with these triggers to determine when the infant was looking at each stimulus. Thus, it was possible to assess the specific hemodynamic changes in each infant individually for each of the stimuli presented. When there were no eye tracking recordings for some of the stimuli presented, the hemodynamic data for these specific stimuli were discarded to ensure that the hemodynamic data evaluated corresponded to the infant’s interest in the stimulus presented, avoiding confounding biases.

### 2.3. Post-Collection with Guidelines for Data Pre-Processing and Analysis

#### 2.3.1. Near-Infrared Functional Spectroscopy Data

An open-source Python package was used for fNIRS analysis (MNE-Python version 1.2) [36]. The raw data were measured in light intensity and converted to optical density. The channels were checked to eliminate optodes that were less than one centimeter apart, and optodes with hemodynamic data lower than 0.5 of the scalp coupling index (SCI).

Then, the data were converted from optical density to hemoglobin with the modified Beer-Lambert law [36], which estimates the change in light attenuation proportionally to the concentrations of tissue chromophores oxy- and deoxyhemoglobin, using the partial pathlength factor for each wavelength for light scattering within a tissue. Thus, the modified Beer-Lambert law in MNE includes a scattering term to account for the light that is scattered instead of being absorbed. This makes it more suitable for analyzing light absorption in tissues and other heterogeneous media where scattering is a significant factor.

**Figure 5:**
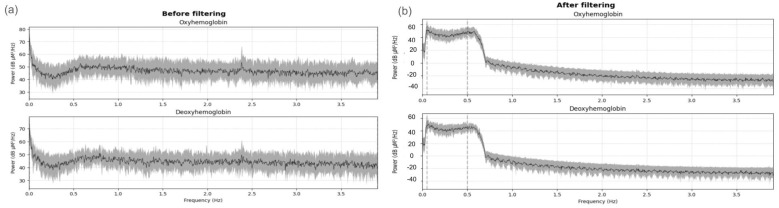
Power spectrum of (**a**) oxyhemoglobin and deoxyhemoglobin signals before filtering. The presence of noise is observed, especially at frequencies around 2.4 Hz. (**b**) Power spectrum of oxyhemoglobin and deoxyhemoglobin signals after applying a band-pass filter (0.05–0.5 Hz). The filter removed high-frequency components and interferences around 2.4 Hz, preserving the frequency bands of interest.

fNIRS neural response data typically have frequencies below 0.5 Hz. A band-pass filter with cutoffs between 0.05 and 0.5 Hz was applied to remove heartbeat artifacts, commonly found between 1 Hz and 3 Hz (e.g., infant heartbeats 60 to 180 bpm) (Figure 5). A baseline correction was performed. Time intervals and rejection criteria were determined based on the maximum peak-to-peak signal amplitude with a threshold of 80 μMolar. In addition, the initial and final time intervals were determined in seconds relative to the time-locked event (tmin = −5; tmax = 12).

**Figure 6:**
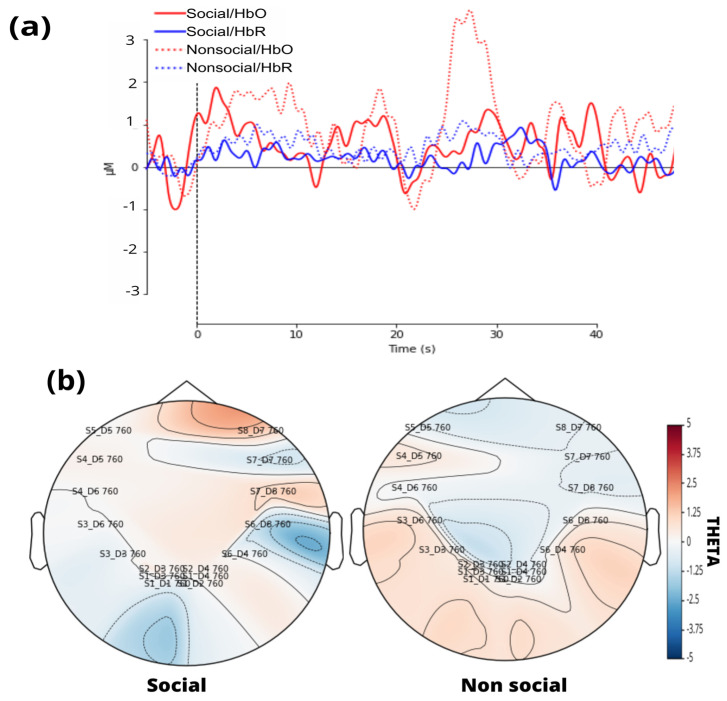
(**a**) Variation in oxyhemoglobin (HbO) and deoxyhemoglobin (HbR) concentration over time in social and non-social conditions. (**b**) Topographic maps represent the distribution of theta coefficient for each condition, highlighting greater activation in the social condition in the visual cortex.

A standard visual representation for fNIRS data was developed, plotting oxygenated (HbO) and deoxygenated (HbR) hemoglobin to illustrate their relationship (Figure 6a). Additionally, a general linear model (GLM) was applied, followed by a visualization of the topographic theta distribution for each stimulus (Figure 6b). Finally, GLM results were extracted, including the theta coefficient for HbO data from each channel.

**Figure 7:**
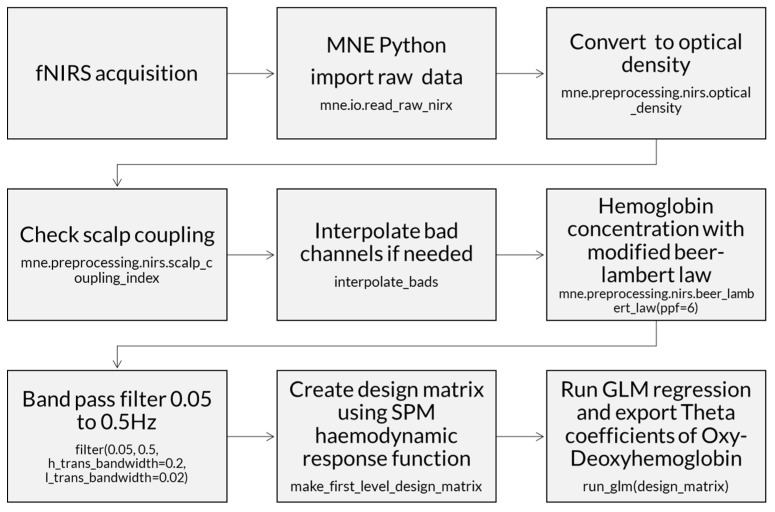
Diagram of fNIRS signal processing, from the collection of fNIRS data to obtaining theta coefficients with the main scripts. fNIRS data were acquired with NIRSLab acquisition software. MNE Python imports raw data and converts to optical density. A scalp coupling index evaluation keeps channels with an index >0.5. Channels with index <0.5 are marked as bad channels, and an interpolation of nearest channels may be needed. Optical density data were converted to hemoglobin concentration with a modified Beer–Lambert law using a partial pathlength factor. Modified Beer–Lambert law includes a scattering term to account for light that is scattered instead of being absorbed. Hemoglobin data were band-pass-filtered, rejecting very low (<0.05 Hz)- and high (>0.5 Hz)-frequency artifacts. A matrix models the expected neural response using the SPM hemodynamic response function. A GLM was fitted for the data and experiment matrix for each channel to extract theta index of HbO and HbR related to each stimulus condition (social, non-social).

Processing of fNIRS data involves several fundamental steps to ensure the quality and accuracy of the measurements performed during the experiments. The following diagram illustrates these steps in a sequential manner, highlighting the main phases involved in the processing of cerebral oxygenation and deoxygenation signals (Figure 7). A supplementary MNE_flow presents the steps to analyze fNIRS data and export the theta index for each channel.

#### 2.3.2. Eye Tracking Data

**Figure 8:**
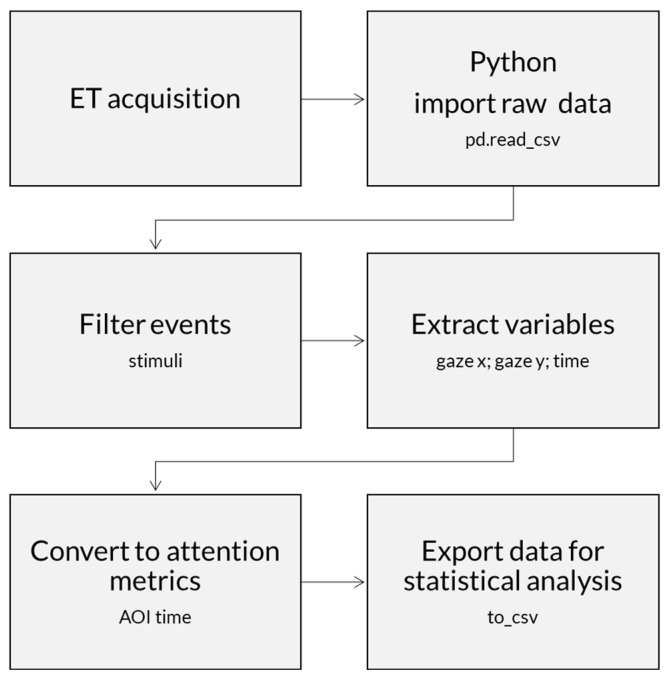
Eye tracking data processing flow. The diagram shows the main steps of the analysis. Raw data are exported from Mangold Vision and imported into Python. Selected events filtered (e.g., according to stimuli and if the infant was looking at the stimulus) and the variables of interest (fixation duration, fixation count, and visit frequency) are selected. An attention index is converted from the variables. Data are exported for statistical analysis.

The data from the ET system were exported to a spreadsheet. A Python script was developed using the Pandas, NumPy, and OS libraries. The script was designed to group and organize the infants’ data efficiently. The extracted variables included fixation duration, fixation count, and frequency of visits to the presented stimulus. The Python script was responsible for structuring the data, ensuring it was ready for subsequent analysis (supplementary ET_flow presents the steps to filter ET data only for valid stimuli with gazes). Additionally, the fixation duration was converted into a percentage of attention by calculating the proportion of time the infant viewed the stimulus relative to the total presentation time. This process allowed for a more accurate analysis of the infant’s visual behavior and interaction with the presented stimulus (Figure 8).

#### 2.3.3. Analysis

Data were summarized using descriptive statistics, expressed as mean, standard deviation, and median for numerical variables. Categorical variables were summarized and expressed as absolute and relative frequencies. The Kolmogorov–Smirnov test was used to determine whether the variables followed a normal distribution. To evaluate the feasibility of the protocol using fNIRS and ET, statistical analyses were conducted focusing on three main aspects: the quality of the collected data, the reliability of the measurements, and the correlation between metrics.

In analyzing the protocol’s success rate, the effectiveness of combining fNIRS and ET was evaluated, taking into account the quality of data collected from both systems. Initially, strict criteria were defined to determine valid data. For fNIRS, data were considered valid if they did not exhibit excessive artifacts, if head movements were minimal, and if there was good sensor coupling. For ET, valid data corresponded to correctly tracked eye data during stimulus presentation and with no data loss. The success rate was then calculated as the proportion of valid attempts in relation to the total number of attempts, allowing for the identification of the protocol’s effectiveness across different age groups.

A Bland–Altman analysis was performed to evaluate the agreement between measurements obtained from both devices, such as fNIRS and ET data. The process involves calculating the difference between the measurements from each system for each data point, followed by the average of these measurements. Based on the differences, the limits of agreement are calculated, indicating the range within which the differences between the two systems are considered acceptable. A Bland–Altman plot is then generated, with the differences plotted on the y-axis and the means on the x-axis. This analysis helps to identify any systematic bias (if the mean of the differences is not zero) and to verify whether the differences between the methods vary with the intensity of the measurements. For fNIRS and ET, this analysis can reveal whether brain oxygenation is related to eye fixation time, thereby helping to validate the agreement between the systems and the efficacy of the experimental protocol. Statistical significance was set at *p* < 0.05. Data analysis was conducted using Statistical Package for the Social Sciences (SPSS^®^), version 23.0.

## 3. Results

The study sample consisted of 43 children at risk for neuropsychomotor development disorders, aged between 1 and 12 months. The mean age was 6.5 months (SD ± 3.2 months). The sample included 25 girls (58%) and 18 boys (42%). The majority of the infants (85%) came from families with a monthly income of up to two minimum wages, reflecting a vulnerable socioeconomic profile.

Initially, all 43 participants completed the experimental protocol and were considered eligible for analysis. Of these, 16 children were between 1 and 6 months old, and 27 were in the 6 to 12 month age range. However, due to the presence of excessive artifacts in the fNIRS signal and the lack of minimal eye fixation during the presentation of the stimuli, 24 participants were excluded from the analysis. Thus, valid data were obtained from 8 infants in the 1-to-6-month-old group and 11 infants in the 6-to-12-month-old group, totaling 19 participants with usable records in both systems. Therefore, the overall success rate of the protocol was approximately 44.2%. Specifically, the success rate was 50% for the 1-to-6-month-old group (8 out of 16 infants) and 40.7% for the 6-to-12-month-old group (11 out of 27 infants).

**Figure 9:**
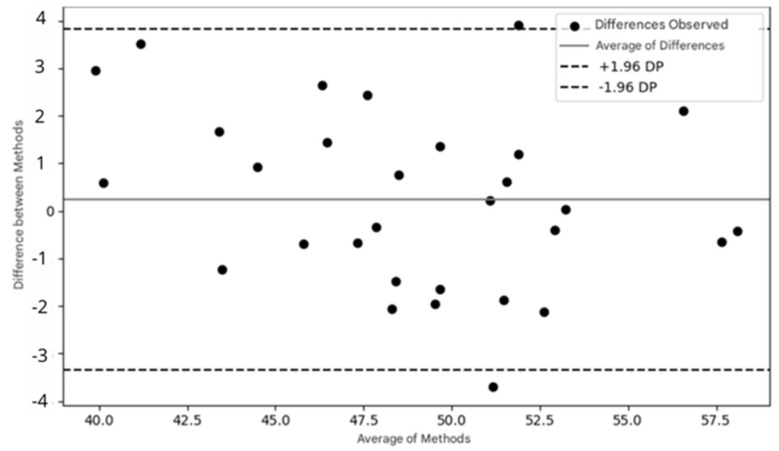
Bland–Altman plot demonstrating the agreement between two measurement methods. The dots represent the observed differences between the methods as a function of the mean of the measurements. The solid line indicates the mean of the differences, while the dashed lines represent the limits of agreement (±1.96 standard deviations of the mean).

A Bland–Altman analysis was performed to identify any systematic bias between fNIRS and ET. The limits of agreement were −4 to 4, suggesting strong agreement between the devices. Additionally, the Bland–Altman plot revealed no systematic bias (40 to 57.5), indicating the variation according to measurement magnitude or intensity. The large limits of agreement may have been influenced by the small sample size (Figure 9).

During the implementation of the proposed protocol, we observed technical challenges and the need to adapt the procedures for clinical use in infants. The main adapted procedures we used to address the methodological challenges were as follows:

1. Difficulty in using a conventional cap: we developed a headband made of soft material that was adjustable to the infant’s head size, and placed it in the waiting room to help them become used to the headband before the experiment.
2. Excessive head movement: we used an infant seat that blocked side vision, used a rolled-up towel or cushion around the neck, providing support and avoiding contact with optodes, and filtered data to correct motion artifacts.
3. Synchronization between fNIRS and ET: we used software that allowed for real-time synchronization of data from both techniques, ensuring that data from both devices were accurately correlated. In addition, we implemented algorithms that could correct synchronization during analysis, aligning temporal data even when small displacements occurred.
4. Infant lower visual acuity: we adjusted the ET parameters to increase flexibility in data capture, allowing tracking even in infants with lower visual acuity.
5. Difficulties with calibrating both devices for different age groups: we used toys that interested the child, and also modified the fixation points by visual animation with figures that were familiar to the child.

Despite the challenges faced during the implementation of these technologies in a clinical environment, we developed a viable and easy-to-apply protocol for young children, with great potential for investigating cognitive aspects.

## 4. Discussion

The combination of ET and fNIRS is a promising approach to investigate neurodevelopment in infants. However, its application still faces significant challenges, especially regarding the standardization of protocols and minimizing interference during data collection [9,18,19].

Our study proposed an innovative protocol that simultaneously uses eye tracking and fNIRS, allowing for a more detailed analysis of the interactions between visual attention and brain activity in infants in the first year of life. This approach aims to overcome methodological limitations, providing greater accuracy in identifying neurodevelopmental patterns.

The success rate obtained in this study, 44.2%, reflects the methodological challenges involved in simultaneously applying functional near-infrared spectroscopy (fNIRS) and eye tracking in infants. The stratified analysis by age group revealed a success rate of 50% in the 1-to-6-month-old group and 40.7% in the 6-to-12-month-old group. These results demonstrate the feasibility of the protocol even in early age ranges and suggest that combined neurophysiological data collection can be conducted with success rates consistent with those reported in the literature.

Previous studies using fNIRS in infants report significant variations in exclusion rates, typically between 30% and 60%, due to factors such as excessive movement, low tolerance to the equipment, and difficulty engaging with stimuli [11,12]. When combined with eye tracking, the inclusion criteria become even more stringent, often resulting in lower success rates [12,37]. Therefore, the percentage obtained in this study aligns with previous findings and reinforces the potential of the proposed protocol.

It is important to note that most studies combining fNIRS and eye tracking are conducted with infants over 6 months of age, when motor control and visual attention are more developed [12,37]. In this context, implementing a protocol that includes infants from an age of 1 month represents a significant methodological advance. By demonstrating that valid data can be obtained in 50% of cases within this age range, this study expands the scope of research on infant neurocognitive development and contributes to the early identification of risk markers in vulnerable populations.

Nevertheless, the results emphasize the importance of continuous methodological adjustments, such as using more visually engaging stimuli, shorter experimental sessions, and more effective algorithms for artifact detection and correction. Improving these aspects can increase success rates and make the combined use of fNIRS and eye tracking even more accessible and informative in clinical and research settings with infants.

To assess the effectiveness and agreement between the two methods, the Bland–Altman plot was used, a widely recognized tool in the literature for verifying the agreement between two measurement methods [38]. The Bland–Altman plot presented in this study shows that most of the points are distributed within the limits of agreement (±1.96 standard deviations of the mean differences), suggesting good agreement between the data collected by fNIRS and eye tracking. This indicates that both methods can be used in combination with confidence.

The relevance of this combination of techniques has been explored in previous research. For example, studies conducted in low- and middle-income countries have assessed the neurocognitive development of infants using these methodologies, highlighting technical challenges for reliable data acquisition. Furthermore, evidence suggests that eye contact influences facial imitation in 4-month-old infants, using fNIRS and EMG to analyze hemodynamic and muscular responses. These findings reinforce both the potential and the challenges of using these tools jointly in understanding child development, justifying the need to improve the protocols to expand their clinical and scientific applicability [9,34].

Brain monitoring technologies in infants at high risk of neurodevelopmental disorders have not been investigated and have not been integrated into clinical practice in low- and middle-income countries [9]. These studies have explored cognitive processes such as visuospatial attention [39], memory processing [40], language [41], motor action comprehension [42], facial processing [43], and the potential use of these technologies in well-equipped laboratories, far from the clinical reality.

Neurodevelopmental monitoring during early childhood is essential for understanding its early determinants and help children reach their maximum potential [7,31]. A systematic review of 84 studies highlights its application in eye tracking, evidencing patterns of sensory specialization and differences in the development of children with neurodevelopmental disorders.

However, standardized protocols addressing methodological aspects to ensure quality data acquisition are lacking in this population. This study proposed a guideline to implement simultaneous ET with cerebral hemodynamic monitoring in infants at high risk for neurodevelopmental alterations in a clinical setting with limited resources. Technical challenges were identified and components and procedures for clinical monitoring were described.

The following methodological challenges to obtain quality ET and fNIRS data were identified: difficulty in calibrating the equipment for different age groups; difficulty in using the conventional cap due to discomfort on the head and below the jaw; excessive movement leading to motion artifacts; low visual acuity; difficulty in obtaining optical signals; interference in the equipment; and scarcity of reference data for interpreting the results.

Studies [35] have shown significant sample losses, poorly planned data analyses, and the need for a well-described methodological analysis [44,45]. Furthermore, previous studies have been from European or African countries [5,46], highlighting the scarcity of protocols for the Brazilian population and hindering reproducibility in regions with limited resources.

Pre-processing and data analysis in infants are challenging, as they require the immediate examination of signals and the adjustment of optode positions, unlike in adults. Establishing a quantitative analysis between individuals is also difficult due to the variability in oxygenation and deoxygenation patterns observed in studies, explained by the relative nature of hemodynamic data [47]. Despite the difficulties, technologies for monitoring visual cognitive function in infants allow for the early detection of neurodevelopmental changes, changes in habilitation services offered to the population, and the dissemination of knowledge among health professionals and users of the Brazilian Unified Health System.

One limitation of this study concerns the selection of visual stimuli used to assess cognitive and attentional responses. The schematic face image was chosen to represent basic facial patterns, while the photograph of an infant was selected due to its potential to activate brain regions associated with empathy and attentional engagement. Although the preliminary analyses indicated that variations between the stimuli did not compromise the validity of the results—since the eye fixation patterns remained consistent in regions of interest—this difference might still influence participants’ processing of the stimuli, potentially introducing bias in data interpretation.

## 5. Conclusions

The proposed protocol demonstrated the feasibility of combining fNIRS and eye tracking for the collection of neurophysiological data in infants, with an overall success rate of 44.2%, extending the application of these methodologies beyond research laboratories. The approach, which includes infants as young as 1 month, represents a significant methodological advancement, broadening the scope of investigation into infant neurodevelopment and enabling the early identification of risk markers in vulnerable populations. Furthermore, the study developed a viable assessment model for longitudinal monitoring of neurodevelopment from the first month of life, providing valuable parameters to understand typical and atypical development in this population and assisting in the early detection of potential dysfunctions.

## Data Availability

The data that support the findings of this study are available upon reasonable request from the corresponding author. Due to ethical and privacy considerations, individual-level data, including raw fNIRS and eye tracking datasets, cannot be publicly shared. Researchers interested in accessing the data for academic and non-commercial purposes may contact the corresponding author via email.

## Supplementary Materials

The following supporting information can be downloaded at: https://www.mdpi.com/article/10.3390/brainsci15050469/s1. The code used to process fNIRS and eye tracking data is available in the supplementary material, allowing reproduction and transparency of the analyses performed in this study.
