# Supplementary material for: Simultaneous Eye Tracking and Cerebral Hemodynamic Monitoring in Infants: A Guide for Pediatric Outpatient Follow-Up
Source: Brain Sci. 2025 Apr 28;15(5):469. doi: 10.3390/brainsci15050469 (PMC12110572; doi:10.3390/brainsci15050469)
Supplement: Supplementary file 1 [file brainsci-15-00469-s001.zip › brainsci-3519537-supplementary.pdf]

MNE_Flow

```
# Mount google drive to access files directly from Colab
from google.colab import drive
drive.mount('/content/drive')

# Install dependencies and libraries
!pip install -U --no-deps git+https://github.com/mne-tools/mne-nirs.git@main
!pip install mne
!pip install nilearn
!pip install mne-nirs
!pip install h5io>=0.1.7
!pip install nilearn>=0.9
import os
import mne
import matplotlib.pyplot as plt
import os.path as op
import numpy as np
import pandas as pd
from itertools import compress
from nilearn.plotting import plot_design_matrix
import mne_nirs
from mne_nirs.channels import get_long_channels, get_short_channels, picks_pair_to_idx
from mne_nirs.experimental_design import make_first_level_design_matrix
from mne_nirs.statistics import run_glm

#Read and load the NIRX data
raw_intensity = mne.io.read_raw_nirx('your_folder_path_with_NIRX_files ', verbose=True)
raw_intensity.load_data()

# Set the duration of all annotations to 5 seconds and rename the stimuli events
raw_intensity.annotations.set_durations(5)
raw_intensity.annotations.rename({'1.0': 'Social', '2.0' : 'NSocial'})

# Convert raw intensity data to optical density
raw_od = mne.preprocessing.nirs.optical_density(raw_intensity)

# Check scalp coupling index for each channel nd mark bad channels with coupling <0.5
sci = mne.preprocessing.nirs.scalp_coupling_index(raw_od)
raw_od.info['bads'] = list(compress(raw_od.ch_names, sci < 0.5))

# Interpolate bad channels using nearest neighbor method
raw_od.interpolate_bads(reset_bads = False, method = dict(fnirs = 'nearest'))

# Convert optical density to hemoglobin concentration using modified Beer-Lambert Law
raw_haemo = mne.preprocessing.nirs.beer_lambert_law(raw_od, ppf=6)

# Bandpass filter to hemoglobin data
raw_haemo = raw_haemo.filter(0.05, 0.5, h_trans_bandwidth=0.2, l_trans_bandwidth=0.02)

# Rejection threshold for hemoglobin signal based on peak-to-peak amplitude
reject_criteria = dict(hbo=80e-6)

# Design matrix for GLM fitting
```

```
design_matrix = make_first_level_design_matrix(raw_haemo, drift_model=None,
high_pass=0.041, hrf_model='spm', stim _dur=5)

# GLM model fit to the filtered hemoglobin data
glm_est = run_glm(raw_haemo, design_matrix)

# Converts GLM results to dataframe and download to local computer
df_cha = pd.DataFrame()
df_cha = glm_est.to_dataframe()
df_cha.to_csv('Name_of_your_file.csv', encoding = 'utf-8-sig')
files.download(' Name_of_your_file.csv')
```

ET_flow

```
# Mount google drive to access files directly from Colab
from google.colab import drive
drive.mount('/content/drive')

# Install dependencies and libraries
import pandas as pd
import numpy as np
import os

# Read and load ET data
df = pd.read_csv('your_folder_path_with_NIRX_files ')
df.columns

# Define screen resolution
screen_width = 2560
screen_height = 1440
center_x = screen_width // 2
center_y = screen_height // 2

#Define valid Area of Interest (AOI)
aoi_width = 1500
aoi_height = 800
x_min = center_x - aoi_width // 2
x_max = center_x + aoi_width // 2
y_min = center_y - aoi_height // 2
y_max = center_y + aoi_height // 2

#Check if gaze is in AOI
df['Na_AOI'] = (
    (df['Gaze X'] >= x_min) &
    (df['Gaze X'] <= x_max) &
    (df['Gaze Y'] >= y_min) &
    (df['Gaze Y'] <= y_max)
)

#Filter only stimuli with gaze in AOI and download to local computer
df['Stimuli'] = df['Name'].where(df['Name'].notnull() & (df['Name'] != ''))
df['Stimuli'] = df['Stimuli'].fillna(method='ffill')
df_na_aoi = df[df['Na_AOI'] == True]
df_na_aoi.to_csv('Name_of_your_file.csv', encoding = 'utf-8-sig')
files.download(' Name_of_your_file.csv')
```
